# Strong Inhibition of Celastrol Towards UDP-Glucuronosyl Transferase (UGT) 1A6 and 2B7 Indicating Potential Risk of UGT-Based Herb-Drug Interaction

**DOI:** 10.3390/molecules17066832

**Published:** 2012-06-05

**Authors:** Yong-Sheng Zhang, Yan-Yang Tu, Xing-Chun Gao, Jun Yuan, Gang Li, Liang Wang, Jian-Ping Deng, Qi Wang, Ru-Meng Ma

**Affiliations:** Tangdu Hospital, Fourth Military Medical University, Xi’an 710038, Shaanxi, China

**Keywords:** celastrol, UDP-glucuronosyltransferase (UGT), herb-drug interaction

## Abstract

Celastrol, a quinone methide triterpene isolated from *Tripterygium wilfordii* Hook F., has various biochemical and pharmacological activities, and is now being developed as a promising anti-tumor agent. Inhibitory activity of compounds towards UDP-glucuronosyltransferase (UGT) is an important cause of clinical drug-drug interactions and herb-drug interactions. The aim of the present study is to investigate the inhibition of celastrol towards two important UDP-glucuronosyltransferase (UGT) isoforms UGT1A6 and UGT2B7. Recombinant UGT isoforms and non-specific substrate 4-methylumbelliferone (4-MU) were used. The results showed that celastrol strongly inhibited the UGT1A6 and 2B7-mediated 4-MU glucuronidation reaction, with 0.9 ± 0.1% and 1.8 ± 0.2% residual 4-MU glucuronidation activity at 100 μM of celastrol, respectively. Furthermore, inhibition kinetic study (Dixon plot and Lineweaver-Burk plot) demonstrated that celastrol noncompetitively inhibited the UGT1A1-mediated 4-MU glucuronidation, and competitively inhibited UGT2B7-catalyzed 4-MU glucuronidation. The inhibition kinetic parameters (Ki) were calculated to be 0.49 μM and 0.045 μM for UGT1A6 and UGT2B7, respectively. At the therapeutic concentration of celastrol for anti-tumor utilization, the possibility of celastrol-drug interaction and celastrol-containing herbs-drug interaction were strongly indicated. However, given the complicated nature of herbs, these results should be viewed with more caution.

## 1. Introduction

**Figure 1:**
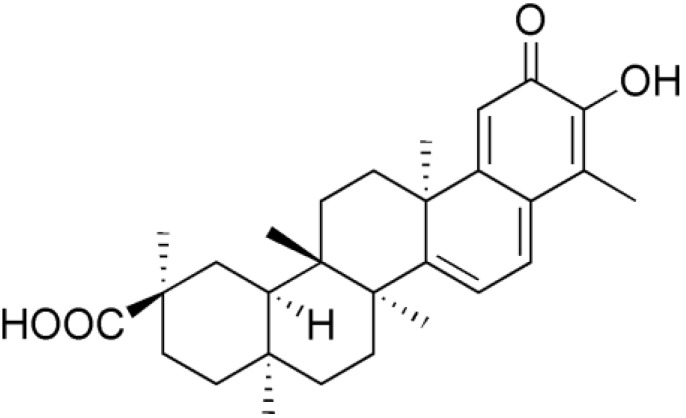
The structure of celastrol.

*Tripterygium wilfordii* Hook F. is an ivy-like vine, widely used in Traditional Chinese Medicine for hundreds of years to treat fever, chills, edema and carbuncles [1]. Celastrol (Figure 1), a quinone methide triterpene, is one of active components isolated from *Tripterygium wilfordii* Hook F. [2]. Celastrol has been widely used to effectively treat autoimmune diseases, chronic inflammation, asthma, and neurodegenerative diseases [3,4,5,6]. Additionally, the anti-tumor activity of celastrol has been drawing much attention [7,8].

Herb-induced adverse effects are a major cause that limits the clinic utilization of herbal medicines. The metabolic behaviour of herbal components plays a key role in herbs’ adverse effects, including metabolic activation-induced herb toxicity and metabolic enzymes inhibition-based herb-drug interactions [9]. For metabolic enzymes inhibition-based herb-drug interactions, inhibition of cytochrome P450 (CYP) by herbal constituents has been widely investigated and regarded as a major reason, because CYPs are involved in the metabolism of most clinical drugs [10]. Additionally, many selective probe reactions of CYPs can be effectively utilized to investigate the inhibition of herbal constituents towards various CYP isoforms. In the past years, these probe reactions have been employed to find many herbal ingredients strongly inhibiting CYP isoforms, including ginsenosides [11], corynoline [12], curcumenol [13], and flavonoids [14].

The human uridine glucuronosyltransferases (UGTs) are phase II conjugation enzymes that can conjugate various endogenous substances and exogenous compounds [15]. Glucuronidation reactions catalyzed by UGTs account for >35% of all phase II drug metabolism [16]. Many important endogenous substances can be conjugated by UGTs, such as bilirubin, steroid hormones, thyroid hormones, bile acids, and fat-soluble vitamins [17,18]. Therefore, more attention should be paid to the inhibition of UGTs by herbal components because the influence of UGT-mediated reactions can result in serious herb-drug interactions. Different from CYPs, limited probe substances have been found for UGT-mediated glucuronidation reaction, which has strongly limited the study of the inhibition of compounds towards UGT activity. Therefore, evaluation of inhibition of compounds towards UGT isoforms was always carried out using recombinant UGTs and nonspecific substrates [19,20,21].

To date, the inhibitory potential of celastrol towards UGT isoforms remains unclear. In the present study, the inhibitory effect of celastrol towards two important UGT isoforms (UGT1A6 and UGT2B7) was investigated. Dixon and Lineweaver-Burk plots were used to determine the inhibition type. In Dixon plot and Lineweaver-Burk plot, we can determine the inhibition kinetic type through evaluating the intersection of the lines in the plots. When the intersection is in the second quadrant and vertical axis for Dixon and Lineweaver-Burk plots respectively, the inhibition type is competitive. When the intersection is in the horizontal axis, the inhibition type is noncompetitive.

## 2. Results and Discussion

**Figure 2:**
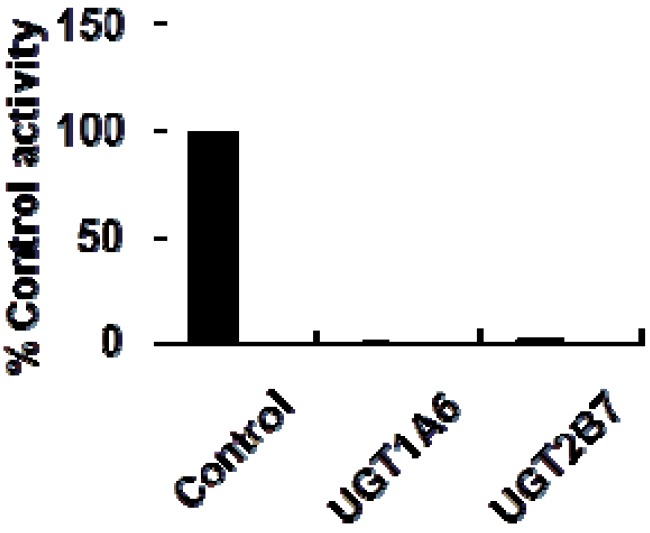
Initial screening of the inhibition of celastrol (100 μM) towards UGT1A6 and 2B7-mediated 4-MU glucuronidation. The experiment was performed in duplicate.

**Figure 3:**
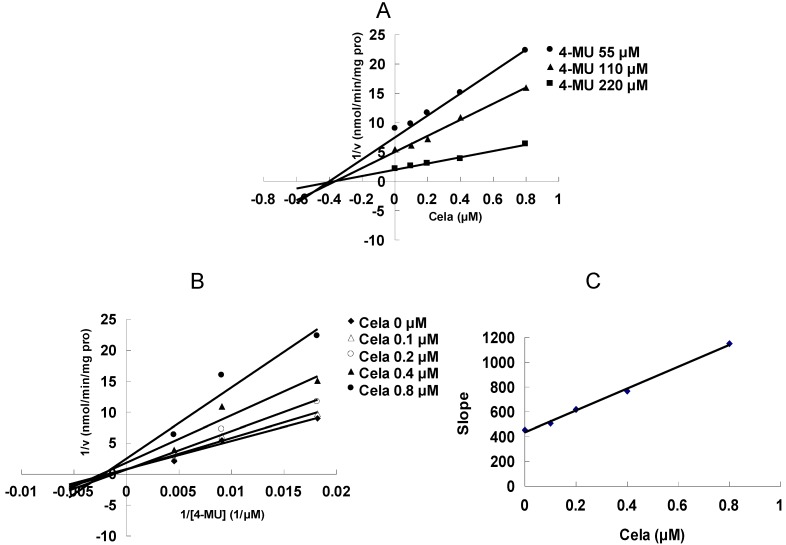
Inhibition kinetic analysis of celastrol (Cela) towards UGT1A6-catalyzed 4-MU glucuronidation. (**A**) Dixon plot of Cela’s inhibition towards UGT1A6-catalyzed 4-MU glucuronidation. (**B**) Lineweaver-Burk plot of Cela’s inhibition towards UGT1A6-catalyzed 4-MU glucuronidation. (**C**) Second plot using slope (obtained from Lineweaver-Burk plot) *vs*. the concentration of Cela.

**Figure 4:**
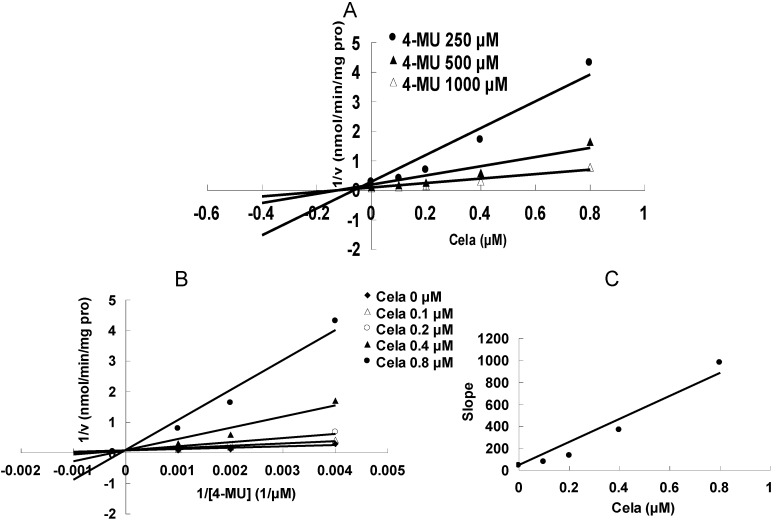
Inhibition kinetic analysis of celastrol (Cela) towards UGT2B7-catalyzed 4-MU glucuronidation. (**A**) Dixon plot of Cela’s inhibition towards UGT2B7-catalyzed 4-MU glucuronidation. (**B**) Lineweaver-Burk plot of Cela’s inhibition towards UGT2B7-catalyzed 4-MU glucuronidation. (**C**) Second plot using slope (obtained from Lineweaver-Burk plot) *vs*. the concentration of cela.

As shown in Figure 2, at 100 μM of celastrol, the residual activity of UGT1A6 and UGT2B7-mediated 4-MU glucuronidation was 0.9 ± 0.1% and 1.8 ± 0.2% of control group. Furthermore, inhibition kinetic analysis was carried out to determine the inhibition type and kinetic parameters. The results showed that celastrol exhibited concentration-dependent inhibitory behaviour towards UGT1A6 and UGT2B7-catalyzed 4-MU glucuronidation. Dixon plot (Figure 3A) and Lineweaver-Burk plot (Figure 3B) showed that celastrol noncompetitively inhibited UGT1A6- mediated 4-MU glucuronidation. The second plot (Figure 3C) using slope (obtained from Lineweaver-Burk plot) *vs*. celastrol concentration showed that the inhibition kinetic parameter (Ki) was 0.49 μM. Different from the inhibition type of UGT1A6, Dixon plot (Figure 4A) and Lineweaver-Burk plot (Figure 4B) demonstrated that the inhibition of UGT2B7 by celastrol best fit the competitive inhibition type, and Ki was calculated to be 0.045 μM (Figure 4C).

UGT1A6 is a major UGT isoform in human liver that glucuronidates various drugs, toxins, and endogenous substrates, including acetaminophen, benzopyrene, and serotonin [22,23]. The alteration of UGT1A6 activity may have important pharmacological, toxicological and physiological consequences. For example, 120-fold variability of UGT1A6 expression in human liver might result in 13-fold variablity in serotonin glucuronidation [24]. UGT2B7, one of the most important UGT isoforms, has been demonstrated to be involved in the glucuronidation of several physiologically important endogenous compounds such as steroid hormones, bile acids, retinoids and fatty acids. Additionally, it can metabolize many drugs including clofibric acid and valproic acid [25]. The glucuronidation of some drugs (e.g., zidovudine, morphine, *etc*.) is exclusively metabolized by UGT2B7.

When celastrol is employed as an anti-tumor agent, the effective dose is approximately 1–10 μM, which is much higher than inhibition concentration of celastrol towards UGT1A1 and UGT2B7. Additionally, as a promising anti-cancer agent, celastrol is likely to be administered with multiple drugs. Therefore, high attention should be given to the celastrol-associated drug-drug interaction due to the inhibition of UGT1A1 and UGT2B7. However, when evaluating the celastrol-containing herbs-drugs interaction, these results should be explained with caution because complicated herbs’ factors might influence the results. For example, processing of herbs and many environmental factors (soil, altitude, seasonal variation in temperature, length of daylight, rainfall patterns, shade) can influence the quantity of celastrol in herbs, which might strongly alleviate the celastrol-containing herbs-drugs interaction. The previous study performed by Du *et al.* showed that many environmental factors (e.g., average precipitation per year, accumulative irradiation time, the soil nitrogen content) could influence the celastrol concentration in *Tripterygium wilfordii* Hook F. [26]. Therefore, these factors should be considered when explaining our present results.

## 3. Experimental

### 3.1. Chemicals and Reagents

Celastrol (purity ≥ 98%) was purchased from Weikeqi Biotechnology Co. Ltd (Sichuan, China). 4-methylumbelliferone (4-MU), 4-methylumbelliferone-β-D-glucuronide (4-MUG), Tris-HCl, 7-hydroxycoumarin and uridine 5'-diphosphoglucuronic acid (UDPGA) (trisodium salt) were purchased from Sigma-Aldrich (St. Louis, MO, USA). Recombinant UGT1A6 and UGT2B7 expressed in baculovirus were obtained form BD Gentest Corp. (Woburn, MA, USA). All other reagents were of HPLC grade or of the highest grade commercially available.

### 3.2. Enzyme Inhibition Experiment

The probe substrate for all the UGT isoforms tested was 4-MU which is a non-selective substrate of UGTs. Incubation with each UGT isoform was carried out as previously reported [25]. The mixture (200 μL total volume) contained recombinant UGTs (final concentration: 0.025 and 0.05 mg/mL for UGT1A6 and UGT2B7) 5 mM UDPGA, 5 mM MgCl_2_, 50 mM Tris-HCl buffer (pH 7.4), and 4-MU in the absence or presence of different concentrations of celastrol. The concentrations of 4-MU were as follows: 110 μM for UGT1A6, and 350 μM for UGT2B7. Celastrol was dissolved in methanol and the final concentration of methanol was 0.5% (v/v). After 5 min pre-incubation at 37 °C, the UDPGA was added to the mixture to initiate the reaction. Incubation time was 120 min for UGT2B7, and 30 min for UGT1A6, respectively. The reactions were quenched by adding 100 μL acetonitrile with 7-hydroxycoumarin (100 μM) as internal standard. The mixture was centrifuged at 20,000 ×g for 10 min and an aliquot of supernatant was transferred to an auto-injector vial for HPLC analysis. The HPLC system (Shimadzu, Kyoto, Japan) contained a SCL-10A system controller, two LC-10AT pumps, a SIL-10A auto injector, and a SPD-10AVP UV detector Chromatographic separation used a C_18_ column (4.6 × 200 mm, 5, Kromasil) at a flow rate of 1 mL/min with UV detection at 316 nm. The mobile phase consisted of acetonitrile (A) and H_2_O containing 0.5% (v/v) formic acid (B). The following gradient conditions were used: 0–15 min, 95–40% B; 15–20 min, 10% B; 20–30 min, 95% B. To evaluate the order of inhibition kinetics and calculate the inhibition parameters, various concentrations of celastrol (0, 0.1, 0.2, 0.4, 0.8 μM) were added to reaction mixtures containing different concentrations of 4-MU (55, 110, 220 μM for UGT1A1; 250, 500, 1,000 μM for UGT2B7). Dixon and Lineweaver plots were adapted to determine the inhibition type, and the second plot of slopes from Lineweaver-Burk plot *vs*. celastrol concentrations was utilized to calculate Ki value.

## 4. Conclusions

Our study indicates the possible occurrence of celastrol-drug interactions or celastrol-containing herbs-drug interactions due to the strong inhibition of celastrol towards UGT1A6 and UGT2B7. However, given the complicated nature of herbs, these results should be viewed with more caution.
